# Counting on the future: fast charge-integrating detectors for X-ray nanoimaging

**DOI:** 10.1107/S1600577523007269

**Published:** 2023-08-23

**Authors:** Junjing Deng, Antonino Miceli, Chris Jacobsen

**Affiliations:** a Advanced Photon Source, Argonne National Laboratory, Lemont, IL 60439, USA; b Department of Physics and Astronomy, Northwestern University, Evanston, IL 60208, USA

**Keywords:** coherent diffraction imaging, fast charge-integrating detectors, X-ray nanoimaging, commentary

## Abstract

A fast charge-integrating detector has been showcased for high-resolution X-ray ptychography. The advancement in developing detectors of this kind, with rapid framing capabilities, holds paramount significance in harnessing the full potential of emerging diffraction-limited synchrotron sources for X-ray nanoimaging.

X-ray nanoimaging is advancing quickly towards and beyond 10 nm resolution by scanning focused coherent beams and then collecting and iteratively phasing far-field diffraction data in a method called ptychography (Fig. 1 ▸). This yields phase and absorption contrast images and tomograms, sometimes with tensor information [such as magnetic field direction (Donnelly *et al.*, 2017 ▸)], beyond the efficiency and resolution limits of X-ray optics.

These rapid advances have been enabled by a steady increase in available coherent X-ray flux, as depicted in the inset of Fig. 1 ▸. Recent and noteworthy advancements have been propelled by the use of multi-bend achromat lattices in electron storage rings. Beyond these synchrotron light sources, X-ray free-electron lasers provide even greater coherent flux, but with such intense and spaced-in-time pulses that one must worry about possible destruction of the sample by the first pulse. Both source types are creating another challenge: photons in detector pixels can arrive so quickly that photon-counting circuitry cannot keep up, as the photon-generated voltage ‘spikes’ become difficult to separate from each other in time.

One viable solution to address this detector challenge is to move from photon counting to charge integration, where one simply measures the total charge accumulated in each pixel during a detector ‘frame’ time. With the knowledge of monochromatic illumination, one can then infer the number of detected photons. This was first done in charge-coupled devices or CCDs, where a voltage sequence was used to move per-pixel charge out to a collecting amplifier and digitizer. Through the implementation of multi-row parallelization, this approach has reached frame rates of around a kilohertz. Another approach has involved the utilization of bump-bonding to attach a sensor to per-pixel charge integration circuitry, sometimes with mechanisms to adjust the analog gain during a detector frame with frame rates increasing to several and even tens of kilohertz. This latter approach of using hybrid pixel array detectors (HPADs) has seen widespread application (and commercial availability) first for photon counting but more recently for charge integration as well. Some variants of these detectors even store charge from each pixel for hundreds of frames at megahertz frame rates, with digitization and data transmission occurring in a burst afterwards.

In this issue of *Journal of Synchrotron Radiation*, researchers (Takahashi *et al.*, 2023 ▸) in Japan report an advance both in per-pixel photon arrival rate and in overall detector frame rate by using a single monolithic CMOS chip which both collects the charge (the sensor in HPADs) and incorporates per-pixel charge integration and digitization circuitry (the application-specific integrated circuit in HPADs). This CMOS approach can potentially offer greater flexibility in the choice of pixel size, and can reduce detector fabrication complexity (and perhaps lead to even faster frame rates, and lower cost) by eliminating relatively high capacitance bump-bonding between two chips with different processing requirements. This detector has been demonstrated for nanocrystal imaging at ESRF, an upgraded fourth-generation synchrotron source, with improved performance compared with a photon-counting detector (Grimes *et al.*, 2023 ▸). As reported in the paper by Takahashi *et al.* (2023 ▸), this detector is now being used for ptychographic imaging at 10 nm resolution over extended objects. A standout attribute showcased by this detector in this paper is its remarkable ability to record a peak intensity of approximately 250 million photons per pixel per second. This rate outperforms that of traditional photon-counting detectors, which typically encounter limitations around 1 million counts per pixel per second (with some correction possible to handle slightly higher rates).

The development of CMOS detectors with charge integration, and with fast-framing capabilities, will likely prove important as X-ray nanoimaging advances along with the development of X-ray sources with increasing coherent flux.

## Figures and Tables

**Figure 1:**
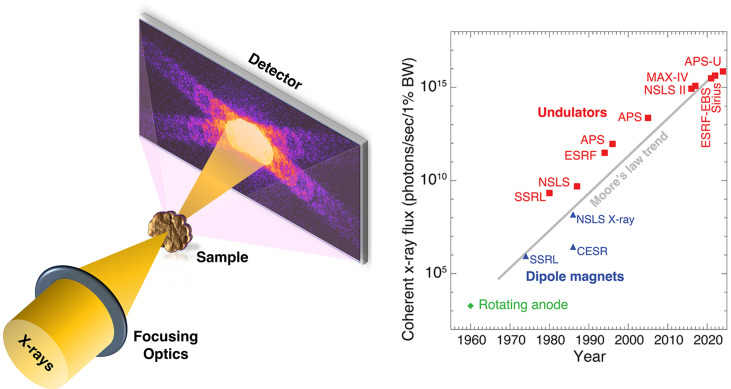
Schematic illustration of X-ray ptychography, where a coherent beam is scanned across the sample. Pixelated detectors record the far-field diffraction pattern, with beyond-optic, high-spatial-resolution information recorded at large scattering angles. This approach benefits tremendously from the historical increase in available coherent flux, which outpaces Moore’s law for integrated circuits. Taking full advantage of these sources requires the development of advanced detectors, such as reported in this issue by Takahashi *et al.* (2023 ▸).
